# Multi-Laboratory Evaluation of Modified BAM Chapter 19b and Long-Term Detection of *Cyclospora cayetanensis* in Fresh Salsa/Pico de Gallo

**DOI:** 10.3390/microorganisms14091980

**Published:** 2026-09-08

**Authors:** Sonia Almeria, Ellie Lauren Rogers, Jeremi Mullins

**Affiliations:** 1 Virology and Parasitology Branch, Division of Food and Environmental Safety (DFES), Office of Applied Microbiology and Technology (OAMT), Office of Laboratory Operations and Applied Sciences (OLOAS), Human Foods Program (HFP), Food and Drug Administration (FDA), Department of Health and Human Services (DHHS), 8301 Muirkirk Road, Laurel, MD 20708, USA; ellielaurenrogers@gmail.com; 2 Joint Institute for Food Safety and Applied Nutrition (JIFSAN), University of Maryland, College Park, MD 20742, USA; 3 Division of Science Program Coordination, Office of Regulatory Testing & Surveillance (ORTS), Office of Laboratory Operations and Applied Science (OLOAS), Human Foods Program, Food and Drug Administration, Department of Health and Human Services, 60 Eighth Street, N.E., Atlanta, GA 30309, USA; jeremi.mullins@fda.hhs.gov

**Keywords:** *Cyclospora cayetanensis*, BAM Chapter 19b modifications, evaluation study, long-term detection, *Cox3* gene, fresh salsa/pico de gallo, qPCR

## Abstract

*Cyclospora cayetanensis* is a foodborne parasite that causes the human diarrheal disease cyclosporiasis. Multiple outbreaks of *C. cayetanensis* have been linked to Mexican-style dishes containing uncooked cilantro. The Bacteriological Analytical Manual (BAM) Chapter 19b method for the detection of *C. cayetanensis* has been validated in cilantro, but not in complex foods containing cilantro, such as fresh salsa/pico de gallo. In a previous study, specific modifications of the BAM method were performed in fresh salsa/pico de gallo for optimal detection of *C. cayetanensis*. In the present study, further evaluation of those modifications in fresh salsa/pico de gallo were performed using the updated BAM Chapter 19b mitochondrial-based qPCR (Mit1C qPCR). Six FDA laboratories analyzed six blind-coded salsa/pico de gallo test samples each. These samples included two unseeded samples, three samples seeded with five oocysts, and one sample seeded with 200 oocysts. All laboratories performed the updated washing and DNA extraction modification steps. The overall *C. cayetanensis* detection rates across laboratories for fresh salsa/pico de gallo samples inoculated with 200 oocysts, five oocysts, and un-inoculated samples were 100% (6/6), 100% (18/18), and 0% (0/12), respectively. The method showed extremely high sensitivity and high reproducibility across all laboratories. Un-inoculated samples were all negative for each laboratory, indicative of high *C. cayetanensis* specificity. This is the first evaluation of the performance of detection of *C. cayetanensis* using the updated BAM Chapter 19b (Mit1C qPCR) in a complex matrix, e.g., fresh salsa/pico de gallo. A second study evaluated the presence of the parasite in a long-term study in fresh salsa/pico de gallo samples inoculated with *C. cayetanensis* and analyzed up to three weeks after inoculation. Samples were positive for the detection of the parasite at each time-point of the study, indicating that the method could be used to detect the parasite in leftovers of fresh salsa/pico de gallo up to three weeks old in outbreak investigations. Evaluation and deploying effective methods to detect *C. cayetanensis* in high-risk fresh produce and prepared dishes are critical to monitor and respond to active outbreaks.

## 1. Introduction

*Cyclospora cayetanensis* is an environmentally persistent foodborne parasite, responsible for many outbreaks and clinical cases in the U.S. and Canada since the 1990s. Infection occurs from contaminated fresh produce consumption, notably, in foods/dishes that are eaten uncooked [1,2,3,4]. Although the disease is self-limiting in most immunocompetent patients, it may be presented as severe, protracted, or chronic diarrhea in immunocompromised people, which can lead to hospitalization [5,6]. Hundreds of thousands of environmentally persistent oocysts may be shed per diarrheal episode. As a human-specific parasite, *C. cayetanensis* contamination is especially problematic in fresh produce growing and handling facilities.

Multiple outbreak clusters of *C. cayetanensis* have been traced back to the consumption of dishes in Mexican-style restaurants related to contaminated cilantro [7,8,9]. From 2013 to 2015, U.S.-based outbreaks were linked to imported cilantro from Puebla, Mexico [1,7,10]. In 2013, 631 cases of cyclosporiasis were identified in 25 states, with one cluster traced back to a Mexican-style restaurant in southeastern Texas. Several prepared and served salsas at this restaurant all contained fresh cilantro, the most probable agent of infection [7]. In 2018, three cyclosporiasis clusters were associated with Mexican-style restaurants in the Midwest, resulting in 53 confirmed illnesses. Additional clusters were identified in multiple other states; however, the source and agent(s) of contamination remain undetermined [1,8]. More recently, 47 cases of cyclosporiasis were associated with consumption of food from a Mexican-style restaurant in Alabama. Epidemiologic, laboratory, environmental, and traceback evidence from this outbreak linked the illness to contaminated cilantro imported from Mexico [9].

Mexican-style dishes such as salsa have been increasing in popularity in many countries, including the U.S, where salsa has outsold ketchup since 1991 [11]. Fresh salsa/pico de gallo is an uncooked dish made from chopped raw ingredients, including tomatoes, onion, cilantro, fresh peppers (jalapeño, bell, serrano), lime juice or vinegar, and salt. As an uncooked product containing known *C. cayetanensis* vectors like cilantro, pico de gallo is considered a high-risk food commodity for *C. cayetanensis* infections and outbreaks in North America. Complex dishes containing uncooked produce are especially problematic in outbreak investigations due to the difficult nature of identifying and tracing the agent of contamination. As a result, many *C. cayetanensis* outbreaks are underreported, highlighting the need for highly accurate detection and surveillance methods.

*Cyclospora cayetanensis* presents unique challenges for research and detection as *C. cayetanensis* oocysts cannot be enriched or cultured in vivo or in vitro. Only clinical samples from the stool of infected patients can be used as sources of oocysts, which are extremely limited in practice. Low levels of oocysts are expected in fresh produce samples, but as few as one to ten oocysts could induce cyclosporiasis. Thus, molecular methods with high levels of specificity and sensitivity are needed to ensure efficient and effective detection. The current method for the detection of *C. cayetanensis* in fresh produce, published in the Bacteriological Analytical Manual (BAM), Chapter 19b, is a molecular detection method utilizing real-time polymerase chain reactions (PCR) [12]. This method was originally validated for the high-risk commodities of cilantro, raspberries, and Romaine lettuce by single laboratory validation (SLV) studies, which showed that the qPCR method is primer-/probe-specific, and there is no cross-reaction with other closely related parasites [13] and in Romaine lettuce by multi-laboratory validations (MLV) [14]. The method has also been validated in parsley, basil, and broccoli [15], and can be effectively used for fresh produce which have been linked to *C. cayetanensis* outbreaks. To date, no multi-laboratory evaluation has been performed for *C. cayetanensis* detection in any complex matrix containing one or multiple of these agents of infection. Accordingly, it is largely unknown whether other ingredients will hinder *C. cayetanensis* detection in these commodities of concern.

The current BAM Chapter 19b method includes a wash step, in which oocysts are recovered from the produce, followed by DNA extraction from the concentrated wash pellets, and a dual TaqMan PCR assay. Primers and probes are specific for the mitochondrial Cox3 gene of *C. cayetanensis* and for an internal amplification control (IAC) to monitor possible matrix-based reaction inhibition, using the PrimeTime^TM^ Gene Expression Master Mix (IDT technologies, Coralville, CA, USA). While the method has been validated for determining the presence of *C. cayetanensis* oocysts in cilantro alone, detecting the parasite becomes more difficult when a suspected ingredient is embedded with multiple other ingredients, such as a fresh salsa/pico de gallo. Detection of 5–10 oocysts has been considered the optimal, and so far, the lowest reliable level of detection for this parasite in one single commodity of fresh produce [16,17,18,19,20]. However, the updated real-time Mit1C protocol in the BAM Chapter 19b method has not yet been evaluated in fresh salsa/pico de gallo. In a previous study of *C. cayetanensis* detection in fresh salsa/pico de gallo, small modifications of the washing protocol were required to enable sensitive and reproducible detection of *C. cayetanensis* (5 oocysts/25 g sample) when fresh salsa/pico de gallo was processed [21].

The first objective of this study aimed to evaluate the performance of the updated real-time Mit1C (Mit1C qPCR) for the detection of the protozoan parasite *C. cayetanensis* in fresh salsa/pico de gallo samples. Efficacy was determined through a multi-laboratory study comprising six FDA laboratories all following the updated modifications [21]. A second objective aimed to investigate the detection limits of long-term storage of *C. cayetanensis* inoculated salsa/pico de gallo samples, emulating typical commercial fresh salsa distribution time periods. The goal of the second study was two-fold: (1) to determine if *C. cayetanensis* could be detected in outbreak investigations of leftovers of fresh salsa/pico de gallo and (2) to determine the effect of aging contaminated fresh salsa/pico de gallo on *C. cayetanensis* detection rates.

## 2. Materials and Methods

### 2.1. Source of C. cayetanensis Oocysts

The Center for Disease Control (CDC) provided a stock of purified *C. cayetanensis* oocysts, stored in 2.5% potassium dichromate, approved by the Institutional Review Board of the FDA (Protocol 15-039 F). All oocysts were obtained from a singular case and singular patient from Indonesia. Microscopy verified that the oocysts were in good condition. As reported by Assurian et al. [22], this main stock was washed and concentrated by centrifugation with a hemocytometer and diluted to 0.85% NaCl, all prior to enumeration. Two stock samples were generated from this dilution; 20 oocysts μL^−1^ and 1 oocyst μL^−1^.

### 2.2. Multi-Laboratorial Verification Study of Blind-Coded Fresh Salsa/Pico de Gallo Samples

#### 2.2.1. Produce Samples and *C. cayetanensis* Inoculation and Preparation by the Coordinating OAMT Laboratory

Fresh salsa and pico de gallo are identical in their ingredients; however, they are labeled arbitrarily depending on the commercial brand. For the purposes of this study, the commodity of interest will be referred to fresh salsa/pico de gallo. Twenty-four to forty-eight hours prior to the inoculation date, commercially branded fresh salsa/pico de gallo tubs were bought at local grocery stores. The main ingredients listed were tomatoes, onions, bell pepper, cilantro, salt, garlic, and lemon juice. All purchased tubs were well within their expiration dates, and stored immediately at 4 °C.

All sample preparation and seeding procedures followed the guidelines of Almeria et al. [21]. Twenty-five-gram samples of fresh salsa/pico de gallo were divided into three groups: unseeded (0 oocysts), low seeding (5 oocysts), and high seeding (200 oocysts). Each 25 g fresh salsa/pico de gallo sample was placed in a 50 mL centrifuge tube. Each tube was spiked using a micropipette with either (1) the low level using 5 μL of the 1 oocyst μL^−1^ stock, (2) the high level using 10 μL of the 20 oocysts μL^−1^ stock, or (3) left untouched. The negative controls (unseeded) were processed identically to the seeded samples throughout the procedure. All samples were aged at 4 °C for 48–72 h prior to shipment.

#### 2.2.2. Collaborating Laboratories

All involved personnel from each of the Office of Regulatory Testing and Surveillance (ORTS) participating laboratories had previous *C. cayetanensis* real-time PCR produce analysis experience. A list of the collaborating FDA laboratories is included below, alphabetically labeled 1–6:

1. FDA Arkansas Human and Animal Food Laboratory (ARHAFL);
2. FDA Denver Human and Animal Food Laboratory (DENHAFL);
3. FDA Irvine Human and Animal Food Laboratory (IRVHAFL);
4. FDA New York Human and Animal Food Laboratory (NYHAFL);
5. FDA San Francisco Human and Animal Food Laboratory (SANHAFL);
6. FDA Seattle Human and Animal Food Laboratory (SEAHAFL).

#### 2.2.3. Processing of Fresh Salsa/Pico de Gallo Samples by the Collaborating Laboratories

Each laboratory participating in the evaluation study (six ORTS labs) received a set of 6 blind-coded fresh salsa/pico de gallo samples. Two samples remained unseeded. Three samples were seeded with 5 *C. cayetanensis* oocysts (low level) and one (1) sample was seeded with 200 *C. cayetanensis* oocysts (high level) for analysis. The samples (fresh salsa/pico de gallo) were shipped with cold packages to the participating laboratories in Saf- T-Pak STP-320 UN 3373 Category B Insulated Shipping System according to the manufacturer’s instructions and shipped via FedEx Priority Overnight shipping. Each collaborating laboratory confirmed to the coordinating laboratory the receipt of the samples in good condition the next day.

The Standard Operation Procedures (SOPs), which included the wash modification and modifications for DNA extraction, the study schedule, and qPCR plate format, were sent in advance to the participating analysts. Analysts had the opportunity to ask questions about the study plan before samples were received during a kick-off call meeting, two weeks before shipping the samples. All laboratories performed the washing step within 1–2 days of receiving the samples. All samples were processed, and all results were submitted to the coordinating laboratory in less than a week.

Two minor modifications were made to minimize tomato tissue degradation. First, the initial produce washing step from the BAM Chapter 19b method for delicate produce (berries) was modified. The 25 g samples of fresh salsa/pico de gallo were transferred from their respective 50 mL tubes to individual BagPage+^TM^ filter bags. All filter bags were placed upright on the Hoefer Red Rocker tray for 5 min and set at 5.0 for 12 rocks per minute [21]. The rest of the wash steps were unmodified as per BAM Chapter 19b. The detergent Alconox^®^ (Alconox, White Plains, NY, USA) at 0.1% solution was able to sufficiently remove oocysts from the surface of the tomato tissue. Sequential centrifugation recovered, pooled, and concentrated the remaining wash debris. This subsequent pellet underwent a DNA extracting using the FastDNA SPIN Kit for Soil in conjunction with a FasPrep-24 Instrument (MP Biomedicals, Santa Ana, CA, USA). If this pellet was greater than 850 μL, a second modification was implemented from BAM Chapter 19c [23]. The 850 μL ≥ pellet was split into two 2 mL FastPrep^®^ lysing tubes. These two replicates were processed independently until the supernatants could be combined in the same SPIN filter provided by the commercial kit.

The real-time PCR method is analyzed as a qualitative method, indicating whether a result is positive or negative. The cut-off C_T_ value must be lower than or equal to 38.0 to be considered positive. This method followed the guidelines of BAM Chapter 19b, including an IAC probe to identify potential false reactions [12]. The Applied Biosystems 7500 Fast Real-Time PCR system (ABI7500) (ThermoFisher Scientific, Waltham, MA, USA) was used in fast mode. All ABI7500 instruments were properly calibrated prior to the experiment. All laboratories used their own real-time PCR reagents. Each experimental run consisted of the following:

1. Two unseeded samples;
2. Three low level samples;
3. One high level sample;
4. One non-template control (no DNA);
5. One positive control with 10^3^ target copies.

The positive control was commercially prepared synthetic gBlocks gene fragments from Integrated DNA Technologies, Coralville, CA, USA. Each sample was run in triplicated and ¼ dilution. Each control was run in triplicate.

Samples were scored positive if at least one out of three replicates of the real-time PCR were found positive for *C. cayetanensis* in undiluted DNA or at the ¼-diluted DNA levels. Following the BAM Chapter 19b instructions, when a sample produced at least one *C. cayetanensis* target replicate reaction with a C_T_ value > 38, OAMT subject matter experts (SMEs) were consulted. Among all six labs, only one sample from two different laboratories each had an exponentially increasing fluorescence signal with a C_T_ value > 38.0. In those cases, the laboratories consulted with the study coordinators. The SMEs considered both samples as negative; therefore, there was no need to retest any samples following SME consultation. Following the BAM Chapter 19b method, a sample is considered inhibited if, on initial testing (or after re-test if required), the sample IAC target is undetermined or produces an average C_t_ value more than 3 cycles higher compared to the non-template control (NTC).

#### 2.2.4. Data Submission to the Coordinating Laboratory and Analysis of the Collaborative Data

An experimental run from a laboratory was considered complete only after the submission of the Applied Biosystems 7500 FAST Real-time PCR instrument-run files’ original data and exported data, and a summary-reporting excel sheet. From this data, the coordinating laboratory SMEs verified whether each lab successfully executed the methodology and met performance parameters. In addition, it was assessed that all three replicates of the positive control were positive and all three replicates of the NTC were negative, before a run could be considered successful.

The acceptance criteria for data evaluation, as approved by an internal FDA validation committee, included the following:

1. All unseeded samples needed to be found negative for acceptance.
2. All samples seeded at the high level must be found positive for acceptance.
3. Samples seeded at the low level may be either positive or negative for acceptance.
4. Controls (NTC, positive control) of the real-time PCR results must be found acceptable according to the guidance provided at “Interpretation of Results” in the link submitted above.

### 2.3. Long-Term Storage and Detection of C. cayetanensis in Samples of Fresh Salsa/Pico de Gallo Samples

#### Produce Samples and *C. cayetanensis* Inoculation in Samples of Fresh Salsa/Pico de Gallo Samples for Long-Term Storage and Detection

Commercial, fresh salsa/pico de gallo, within their expiration dates, were bought at local grocery stores and stored at 4 °C for no more than 24–48 h prior to oocyst inoculation. Of the fresh salsa/pico de gallo samples, 0.5–0.7 g were added in 2.0 mL Fastprep^®^ tubes (MP Biomedicals, Santa Ana, CA, USA). The samples were then inoculated with 5 and 200 oocysts using a micropipette (3 samples at each seeding level). Unseeded samples were included as negative controls and processed together with the seeded samples. Samples were held at 4 °C for 2, 7, 14 and 21 days after inoculation of fresh salsa/pico de gallo, based on the expected shelf-life of commercial fresh salsa/pico de gallo, 7–14 days past their expiration dates.

The DNA extraction procedure was performed directly in the inoculated samples using the FastDNA SPIN Kit for Soil in conjunction with a FastPrep-24 Instrument (MP Biomedicals, Santa Ana, CA, USA) [12] The real-time PCR assay for *C. cayetanensis* and IAC was performed on an Applied Biosystems 7500 Fast Real time PCR System (ThermoFisher Scientific, Waltham, MA, USA) following the BAM Chapter 19b procedure.

### 2.4. Statistical Analysis

Data were analyzed qualitatively, and results were considered either positive or negative. Collaborative study detection rates were calculated for fresh salsa/pico de gallo samples at each inoculation level in each laboratory. The positive detection rate was defined as the number of positive inoculated samples per seeding level compared to all samples on that seeding level. Statistical analysis was performed using non-parametric one-way analysis of variance (ANOVA) and Kruskal–Wallis’s multiple comparisons tests, using GraphPad Software version 11 (GraphPad, San Diego, CA, USA) where *p* < 0.05 indicated a statistical difference.

## 3. Results

### 3.1. Multi-Laboratory Verification

All six collaborating laboratories successfully executed the analysis in fresh salsa/pico de gallo. Sample results analyzed by each laboratory were retained after data evaluation. Every laboratory performed the method following the two modifications included in the protocol (washing modification and DNA extraction modification). There were no issues during sample processing for any of the laboratories.

Individual C_T_ values per sample and the number of positive replicates for each sample reaction are shown from each laboratory respectively. Every experimental run across all laboratories demonstrated negative real-time PCR negative control replicates and positive real-time PCR positive control replicates, with all positive control replicates outputting comparable C_T_ values. Accordingly, no artificial contamination nor false-negative reactions were observed.

All negative samples (12 samples) were negative to the presence of *C. cayetanensis*, while all seeded samples, 200 oocysts (n = 6) and five oocysts (n = 18), were found positive for the presence of *C. cayetanensis*. Two laboratories each found one sample of five oocysts positive at the ¼-diluted DNA, after being undetermined at the undiluted DNA level (Table 1). The overall detection rates for salsa/pico de gallo samples inoculated with 200 and five oocysts and un-inoculated samples across laboratories were 100% (6/6), 100% (18/18) and 0% (0/12), respectively (Table 1). There were no false positive qPCR results for any sample across all laboratories.

Based on the IAC C_T_ values, no inhibition was observed in any of the study samples in any laboratory (Table 1). Out of all the samples analyzed, two samples, one from two different laboratories each, had an exponentially increasing fluorescence signal with a C_T_ value > 38.0. In those cases, the laboratories consulted with the study coordinators. The SMEs considered both samples as negative.

### 3.2. Long-Term Storage Detection of C. cayetanensis Oocysts in Salsa/Pico de Gallo Samples

Every one of the low and high seeding levels of the aged fresh salsa/pico de gallo samples (0.5–0.7 g), kept at 4 °C for up to 3 weeks, were positive for the presence of *C. cayetanensis* at 2, 7, 14, and 21 days post inoculation (dpi) (Table 2).

**Figure 1:**
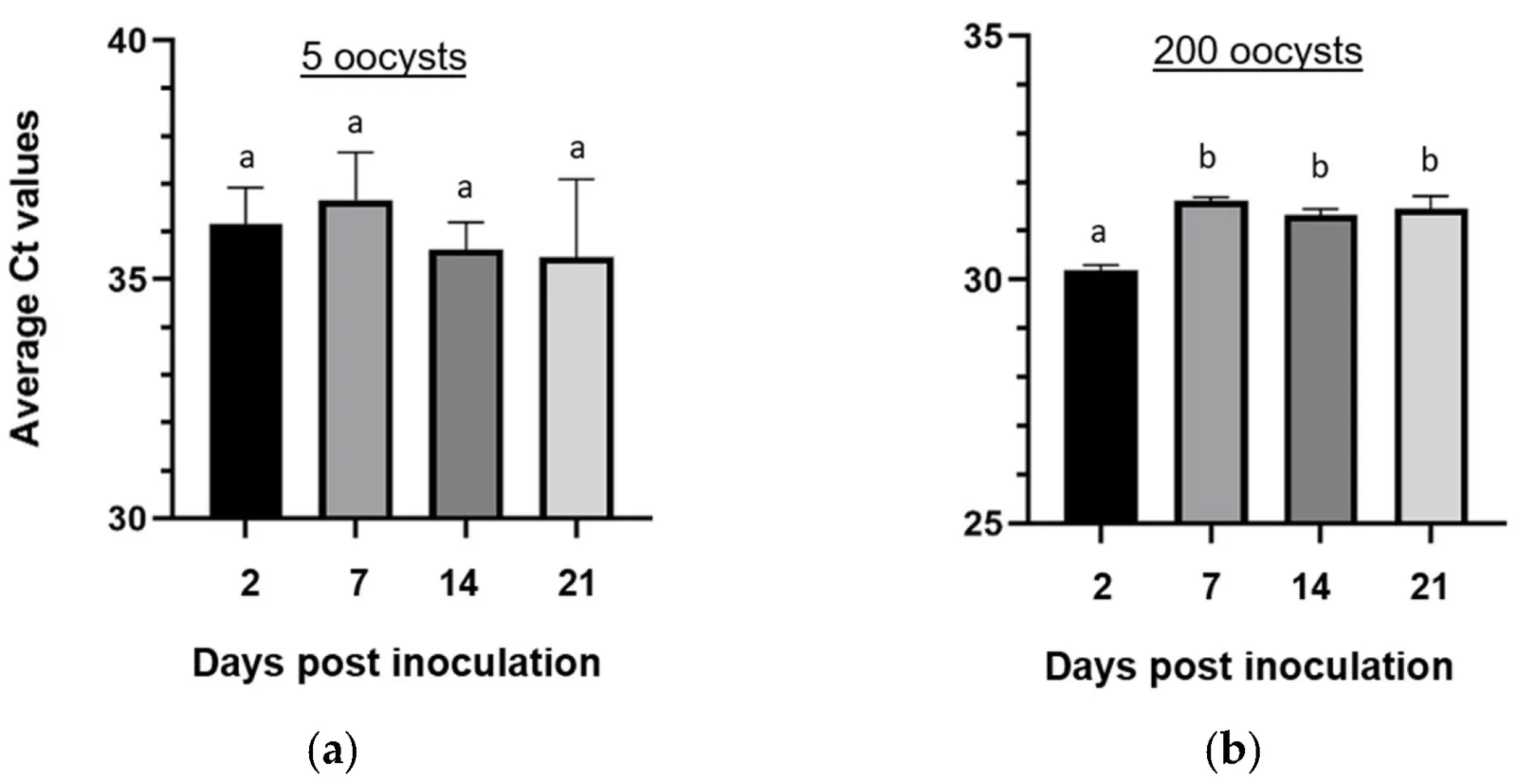
(**a**) Long-term storage of fresh salsa inoculated with 5 *C. cayetanensis* oocysts; (**b**) Long-term storage of fresh salsa inoculated with 200 *C. cayetanensis* oocysts. Different letters (a, b) on top of the columns indicate statistically significant differences among days post inoculation.

A very stable trend in average C_T_ values was observed during the study period by sample collection at the five-oocyst inoculation level (Figure 1a, *p* = 0.533). At the 200-oocyst inoculation level, samples showed an increase in C_T_ values after 2 dpi. Statistically significant lower C_T_ values (*p* < 0.002) were observed at 2 dpi compared to the rest of the samples (Figure 1b). After the decline at 2 dpi, C_T_ values in samples seeded with 200 oocysts remained constant up to 21 dpi. No significant differences were observed among C_T_ values across 7, 14, and 21 dpi in samples seeded with 200 oocysts (Figure 1b).

## 4. Discussion

To efficiently and effectively monitor ongoing cyclosporiasis outbreaks, it is imperative that the mitochondrial target gene (Mit1C) real-time PCR functions equally well across a wide array of fresh-based commodities. This is especially important in complex food matrices such as fresh salsa/pico de gallo, which contain multiple raw ingredients. Considering that many outbreak clusters continue to be traced back to such complex dishes, it is a priority to show that the method works properly across many different commodities, including complex matrices [1,7,8,9,14,21,24]. Additionally, evaluating the efficacy of the method over time, as emulating commercial shipping time periods, is imperative to ensure this method is functional for real-world applications. In this study, a modified real-time PCR method based on the mitochondrial target gene Mit1C was evaluated in the complex food item, fresh salsa/pico de gallo, by six FDA laboratories.

Fresh salsa is a fragile commodity as the tomato tissue degrades readily. A previous study [21] showed that the washing protocol in BAM Chapter 19b required small modifications (a gentle and shortened washing step) to avoid the release of large amounts of debris in the more stringent washing protocol indicated in BAM Chapter 19b. In the present study, even with modified washing, the handling and transportation of fresh salsa/pico de gallo to the collaborating laboratories could have increased the size of washing pellets, and each laboratory reported having more than 850 µL pellets (limit of the size pellets that could be processed in a single DNA tube), requiring a second small modification at the DNA extraction step. This modification is commonly performed during the DNA extraction of agricultural water [23] but was not previously needed when fresh salsa/pico de gallo samples were analyzed at the originating laboratory [21]. These alterations improved *C. cayetanensis* detection in this complex food matrix consistently across all laboratories. As few as five oocysts in 25 g samples were detected, enabling FDA field laboratories to perform and implement this analysis in outbreak investigation samples when fresh salsa/pico de gallo samples are collected. In fact, the six laboratories involved demonstrated proficiency in the execution of the validated method in fresh salsa/pico de gallo. The ease and efficacy of this protocol, as such, enhance the FDA’s capacity to perform regulatory testing of complex matrices such as fresh salsa/pico de gallo for *C. cayetanensis* and to implement the method in this matrix across FDA microbiology laboratories.

The results demonstrated that the modified washing and DNA extraction in this complex matrix allowed the detection of as few as five oocysts using the new Mit1C qPCR for *C. cayetanensis* in fresh salsa/pico de gallo at exceedingly high detection rates (100% in each laboratory and seeding level) and were shown to be highly reproducible among laboratories. In a previous study using the 18S rRNA ribosomal marker as the target in fresh salsa/pico de gallo, samples seeded with five oocysts showed 83.3% of positive detection (n = 12 samples analyzed) [21]. Therefore, the updated mitochondrial target, which was intended for higher specificity, demonstrated improved detection sensitivity. Previous studies showed that the updated mitochondrial method was able to detect five oocysts in raspberries, cilantro, Romaine lettuce, parsley, basil, and broccoli in single-matrix fresh produce [13,14,15]. Detection of as few as five oocysts, the minimum number of reliably inoculated oocysts, was previously observed in complex prepared dishes such as Mexican-style salsas and guacamole [21] using the 18S qPCR. However, to our knowledge, this is the first study to show that the updated Mit1C qPCR method achieved the same low level of detection in a complex matrix. To our knowledge, there are no previous studies on the detection of *C. cayetanensis* or other protozoa parasites in fresh salsa/pico de gallo. Recent studies in some complex fresh produce matrices analyzed the presence of other foodborne protozoa, such as *Toxoplasma gondii, Cryptosporidium* spp., *Giardia duodenalis*, *Entamoeba histolytica* and *C. cayetanensis*, in ready-to-eat mixed salads [25,26,27,28]; *C. cayetanensis* or *Trypanosoma cruzi* in processed açai juice, açai pulp and in sugarcane juices [29,30,31]; and *Cryptosporidium* parvum in self-pressed apple juices [32].

*Cyclospora cayetanensis* has been detected in several single-ingredient fresh produce [reviewed by [33,34]]. More recently, among other fresh produce, the parasite has been found in organic leafy greens and in different types of berries such as strawberries, raspberries, and blueberries [35,36]. The parasite was not found in frozen berries in a study in Italy [37].

All PCR reagents performed as expected across all laboratories, as verified by the positive and negative controls. Likewise, the IAC confirmed there was no matrix-derived inhibition. Furthermore, all the collaborating laboratories demonstrated comparable data compared to the coordinating laboratory, meaning this method is robust and highly reproducible. In addition, no inter-laboratory variations were observed throughout the experiment due to factors such as operator skill, instrument and/or different reagent lot. The coordinating laboratory data were not included in the study comparison as per FDA guidelines. All things considered, this modified real-time PCR assay demonstrated comparable results to previous studies for single-matrix commodities such as cilantro, basil, and parsley [13,14,15].

The new qPCR method showed high specificity in the detection of *C. cayetanensis* in fresh salsa/pico de gallo (all un-inoculated samples were negative) but also showed high sensitivity and high reproducibility in this complex matrix, since all six laboratories achieved 100% detection on the inoculated samples. Two laboratories observed the parasite in one sample each, in the diluted DNA. Lower numbers of oocysts cannot be inoculated in the samples in a reliable manner. The LOD for the MLV of the method has been calculated to be 2.9 oocysts/25 g of fresh produce [14]. Accordingly, inoculating 25 g samples with less than five oocysts would result in unreliable positive/negative results.

Homemade fresh salsa/pico de gallo made from fresh ingredients can last up to three to four days in the refrigerator when stored in an airtight container [38]. The shelf life of commercial fresh salsa can be extended due to the addition of natural (lemon juice, vinegar, or xanthan gum) or artificial preservatives (sodium benzoate, potassium sorbate). Accordingly, the storage time of commercial fresh salsa can vary greatly. In the present study, samples were kept refrigerated and analyzed at intervals for up to three weeks. Complete positive detection was observed at each time-point (2, 7, 14, and 21 dpi), indicating that the method could be used to detect low levels of the parasite in old leftovers of fresh salsa/pico de gallo. Furthermore, the 100% positive detection rates for the low and high seeding levels reaffirm confidence in the ability for this method in long-term *C. cayetanensis* oocyst detection. These results affirm that aged fresh salsa samples can be collected and reliably analyzed in outbreak investigations.

To our knowledge, there are no previous long-term studies regarding *C. cayetanensis* detection in fresh salsa. A previous study analyzed the presence of the parasite in frozen salsa/pico de gallo [21]. In that study, frozen salsa/pico de gallo samples seeded with five *C. cayetanensis* oocysts and held for 4 weeks at −20 °C were positive [21]. A few studies have explored the detection of *C. cayetanensis* oocysts in previously frozen food matrices, such as in dairy products, on basil [39] and on berries [22,40]. It is similarly important to evaluate that the method reliably detects oocyst DNA in older samples that have been refrigerated, rather than frozen. As cyclosporiasis symptoms do not appear immediately due to a median incubation period of around seven days [4,21,41], frozen or old leftovers may be needed for analysis to confirm a link between reported illnesses and food products of concern. The present study confirmed that in addition to frozen samples, the parasite can be detected in low numbers (as few as five oocysts) in aged fresh salsa/pico de gallo, up to three weeks after being inoculated with the parasite, one to two weeks after the expiration date.

The overall goal of switching to a Mit1C target from the previous 18s rRNA target was to improve detection specificity and IAC consistency [13,14,15]. The high degree of specificity, sensitivity, and reproducibility demonstrated throughout this study verify the benefits of using the Mit1C target, especially in complex food matrices. The main limitation of all current *C. cayetanensis* molecular assays is the inability to differentiate live and dead oocysts. Although the presence of oocysts, live or dead, indicates human fecal contamination, rendering the produce unacceptable for human consumption, this relays an inability to quantify infectivity rates. Future studies would be needed to evaluate strategies, such as vital dyes to selectively detect DNA from living cells or analyze gene expression induction [42], to be able to assess the viability of oocysts to more accurately predict the parasite’s infectivity. Although time and monetary costs have been optimized to the best of the current technological standard, the expense of advanced equipment and trained personnel can limit the use of such molecular methods in low-resource countries and laboratories.

## 5. Conclusions

The present interlaboratory evaluation study demonstrated that minor modifications in BAM Chapter 19b to the wash and DNA extraction steps in fresh salsa/pico de gallo improved the parasites’ limit of detection. As few as five oocysts in 25 g of fresh salsa were consistently and reliably detected by all six laboratories involved. In addition, the present study showed that the updated real-time Mit1C probe (Mit1C qPCR) in Chapter 19b was extremely sensitive for the detection of *C. cayetanensis* in this complex matrix. Furthermore, low levels of *C. cayetanensis* oocysts were detected in samples of fresh salsa/pico de gallo up to three weeks post inoculation. This study, therefore, concludes that the Mit1C qPCR method is an effective analytical tool for the detection of *C. cayetanensis* in fresh salsa/pico de gallo, including old leftovers. Additional complex matrices would need to be evaluated in the future to ascertain where modifications may be necessary. The use of the improved detection method for complex food will facilitate our understanding of the presence of *C. cayetanensis* in fresh produce and in the environment. By improving our understanding of *C. cayetanensis* epidemiology and detection, more informed decisions can be made to identify the necessary preventative control measures and reduce foodborne exposure to *C. cayetanensis*.

## Figures and Tables

**Table 1:** Multi-laboratory detection of *C. cayetanensis* in fresh salsa/pico de gallo samples seeded at different seeding levels (0, 5, and 200 oocysts/25 g fresh salsa/pico de gallo) using Mit1C qPCR (BAM Chapter 19b, 2025) by laboratory.

			Mit1C		IAC	
Laboratory No.	Sample	No. Oocysts	Average C_T_	St Dev C_T_	No. Positive Replicates	Average C_T_ ± St Dev
1	V-2	0	Und	Und	0	23.37 ± 0.03
	V-5	0	Und	Und	0	23.36 ± 0.05
	V-3	5	35.3	0.9	3	23.37 ± 0.03
	V-4	5	36.4	0.2	3	23.33 ± 0.02
	V-6	5	35.6	0.4	3	23.33 ± 0.00
	V-1	200	32.0	0.5	3	23.37 ± 0.06
2	V-2	0	Und	Und	0	26.68 ± 0.01
	V-5	0	Und	Und	0	26.65 ± 0.03
	V-3	5	34.4	0.9	3	26.68 ± 0.01
	V-4	5	34.6	0.7	3	26.66 ± 0.01
	V-6	5	36.0	0.5	3	26.60 ± 0.01
	V-1	200	30.6	0.2	3	26.71 ± 0.02
3	V-2	0	Und	Und	0	24.59 ± 0.01
	V-5	0	Und	Und	0	24.61 ± 0.01
	V-3	5	34.9	0.3	2	24.59 ± 0.02
	V-4	5	35.7	NA	1	24.55 ± 0.03
	V-6	5	37.3	0.3	3	24.56 ± 0.03
	V-1	200	29.1	0.1	3	24.65 ± 0.02
4	V-2	0	Und	Und	0	24.67 ± 0.02
	V-5	0	Und	Und	0	24.69 ± 0.01
	V-3	5	36.9	1.4	2 (1/4 dilution)	24.63 ± 0.01
	V-4	5	37.1	0.4	3	24.67 ± 0.00
	V-6	5	36.9	1.5	2	24.68 ± 0.02
	V-1	200	30.6	0.2	3	24.71 ± 0.01
5	V-2	0	Und	Und	0	31.48 ± 0.08
	V-5	0	Und	Und	0	31.42 ± 0.03
	V-3	5	34.9	0.9	3	31.55 ± 0.14
	V-4	5	34.5	0.8	3	31.61 ± 0.04
	V-6	5	32.9	0.7	3	31.82 ± 0.16
	V-1	200	30.3	0.2	3	31.46 ± 0.12
6	V-2	0	Und	Und	0	27.32 ± 0.07
	V-5	0	Und	Und	0	27.06 ± 0.07
	V-3	5	37.0	NA	1	27.00 ± 0.05
	V-4	5	37.5	0.6	2	27.12 ± 0.04
	V-6	5	36.5	1.3	2 (1/4 dilution)	29.90 ± 0.11
	V-1	200	32.5	0.4	3	27.03 ± 0.01

Und: Undetermined (not detected after 40 cycles). NA: Not applicable.

**Table 2:** Long-term detection of *C. cayetanensis* oocysts inoculated in fresh salsa/pico de gallo samples up to 21 dpi.

Days Post Inoculation	Oocyst No.	No. Positive Replicates	Average Mit1C C_T_ ± St Dev	Average IAC C_T_ ± St Dev
2	5	3	36.5 ± 1.2	25.1 ± 0.1
	5	3	35.3 ± 0.2	25.2 ± 0.2
	5	3	36.7 ± 0.5	25.3 ± 0.3
	200	3	30.3 ± 0.2	25.3 ± 0.1
	200	3	30.2 ± 0.1	25.2 ± 0.2
	200	3	30.1 ± 0.1	25.3 ± 0.2
7	5	1	36.0	24.6 ± 0.2
	5	1	37.8	24.7 ± 0.1
	5	3	36.2 ± 1.1	24.6 ± 0.3
	200	3	31.6 ± 0.2	24.6 ± 0.2
	200	3	31.7 ± 0.2	24.8 ± 0.2
	200	3	31.6 ± 0.2	24.7 ± 0.1
14	5	3	35.0 ± 0.9	25.0 ± 0.2
	5	3	36.1 ± 0.6	25.3 ± 0.3
	5	3	35.8 ± 0.8	25.0 ± 0.1
	200	3	31.2 ± 0.6	24.9 ± 0.1
	200	3	31.4 ± 0.3	24.8 ± 0.2
	200	3	31.4 ± 0.6	24.5 ± 0.1
21	5	3	37.3 ± 0.9	24.67 ± 0.1
	5	3	34.9 ± 0.6	24.8 ± 0.6
	5	3	34.2 ± 0.8	25.13 ± 0.3
	200	3	31.7 ± 0.6	24.87 ± 0.1
	200	3	31.2 ± 0.3	24.78 ± 0.2
	200	3	31.5 ± 0.6	24.54 ± 0.1

## Data Availability

The original contributions presented in this study are included in the article. Further inquiries can be directed at the corresponding author.
